# Proprotein convertase subtilisin/kexin type 9 inhibitors protect against contrast-associated acute kidney injury in patients with atherosclerotic cardiovascular disease

**DOI:** 10.3389/fcvm.2024.1384523

**Published:** 2024-07-11

**Authors:** Yu Ma, Hui Fan, Wei Mi, Jing Ma, Yong Deng, Yijie Song, Ximing Li

**Affiliations:** ^1^ Department of Cardiology, Chest Hospital, Tianjin University, Tianjin, China; ^2^ Tianjin Key Laboratory of Cardiovascular Emergency and Critical Care, Tianjin Municipal Science and Technology Bureau, Tianjin, China; ^3^ Clinical School of Thoracic, Tianjin Medical University, Tianjin, China; ^4^ Department of Pharmacy, Chest Hospital, Tianjin University, Tianjin, China; ^5^ Tianjin Institute of Cardiovascular Diseases, Chest Hospital, Tianjin University, Tianjin, China; ^6^ Network Management Center, Chest Hospital, Tianjin University, Tianjin, China

**Keywords:** evolocumab, contrast-associated acute kidney injury, percutaneous coronary intervention, propensity-score matching, PCSK9

## Abstract

**Background and aims:**

Contrast-associated acute kidney injury (CA-AKI) may occur in patients undergoing medical procedures involving x-rays and radiocontrast media, potentially resulting in prolonged renal impairment. However, no effective treatments are available. Therefore, this study aimed to investigate the efficacy of evolocumab, a proprotein convertase subtilisin/kexin type 9 inhibitor, in reducing CA-AKI incidence among patients with atherosclerotic cardiovascular disease (ASCVD) undergoing percutaneous coronary intervention.

**Methods:**

This retrospective cohort study included patients who underwent percutaneous coronary intervention between January 2020 and December 2021 at Tianjin Chest Hospital. The study endpoint was CA-AKI incidence, and the impact of selection bias and other potential confounding factors was mitigated using bias matching. Overall, 1,642 patients were included in this study: 821 patients received evolocumab treatment before contrast agent application, and 821 did not receive such treatment.

**Results:**

CA-AKI incidence was 6.21% and 8.04% in the evolocumab and control groups, respectively. After propensity-score matching, the incidence rate was 5.09% and 14.16% in the evolocumab and control groups, respectively. Evolocumab treatment significantly reduced CA-AKI incidence (*p *< 0.001). Consistent findings were obtained in the subgroups of individuals with type II diabetes mellitus, chronic heart failure, and hypertension. Evolocumab exhibited a significantly greater protective effect in the high- and extremely high-risk populations than in the low- and middle-risk populations (*p *< 0.001).

**Conclusions:**

Evolocumab administration significantly reduced CA-AKI incidence among patients with ASCVD. Notably, this effect was more prominent within the subset of high- and extremely high-risk individuals who were already experiencing CA-AKI.

## Graphical Abstract

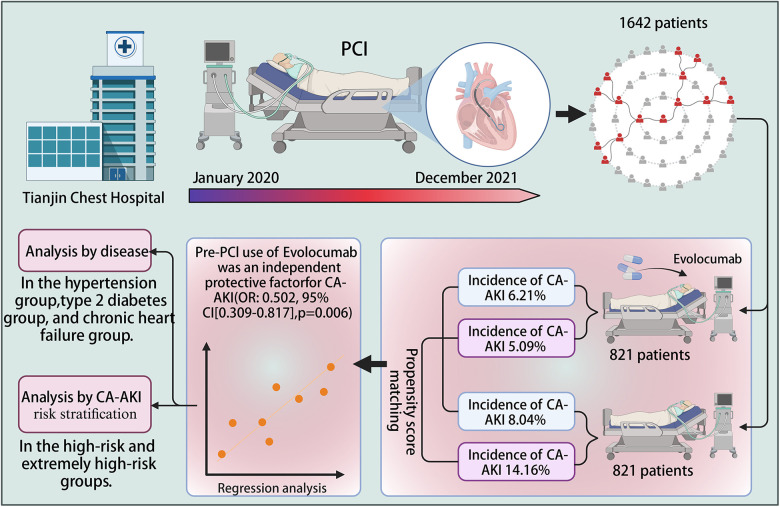

## 1 Introduction

The global implementation of medical procedures involving x-rays and radiocontrast media has significantly increased. Nevertheless, iodinated contrast media can lead to acute kidney injury, known as contrast-associated acute kidney injury (CA-AKI), manifesting within days after its administration. CA-AKI may result in prolonged renal impairment, necessitating renal replacement therapy and contributing to increased mortality. However, no established treatment for CA-AKI is currently available; therefore, its prevention has become a priority when using such procedures (1).

CA-AKI pathophysiology might be based on oxidative stress, inflammatory processes, and programmed cell death; however, the exact mechanism of its development remains unknown. The contrast agent's inherent toxicity can induce conditions such as medullary ischemia or hypoxia. Consequently, these conditions promote the generation of oxygen radicals that damage the renal tubular epithelial cells. Native inflammatory factors are also released when cell necrosis occurs. This overwhelming aseptic inflammation compromises the renal tubular epithelial cells, further exacerbating cellular oxidative stress. Moreover, this escalation causes cell necrosis, creating a detrimental feedback loop (2).

Proprotein convertase subtilisin/kexin type 9 (PCSK9) inhibitors are emerging lipid-lowering agents that protect against atherosclerotic cardiovascular disease (ASCVD) by reducing low-density lipoprotein (LDL) cholesterol levels (3, 4). Recent clinical studies, including the Evolocumab for Early Reduction of LDL Cholesterol Levels in Patients With Acute Coronary Syndrome and Evolocumab in Acute Coronary Syndrome trials, have validated the beneficial impact of pre-percutaneous coronary intervention (PCI) use of PCSK9 inhibitors in patients with ASCVD, demonstrating an improved patient prognosis, reduced incidence of major adverse cardiovascular events, and decreased overall mortality rate. The findings also recommend the early application of PCSK9 inhibitors in patients with acute coronary syndrome (5–8).

The multifaceted impact of PCSK9 inhibitors is notably gaining substantial research attention. PCSK9 inhibitors may potentially participate in the regulation of multiple pathophysiological processes, encompassing inflammatory response, oxidative stress response, programmed cell death, and so forth, exerting a protective effect on ASCVD (9). The pathogenesis of CA-AKI is likewise associated with the activation of inflammatory responses, oxidative stress, apoptosis, and pyroptosis. Consequently, we hypothesize that PCSK9 inhibitors may exert a protective effect on CA-AKI by inhibiting the aforementioned reaction processes. Furthermore, a previous study has demonstrated the efficacy of combination therapy, comprising PCSK9 inhibitors, hydration, and statins, in preventing CA-AKI among patients with acute myocardial infarction (10).

Therefore, this study aimed to investigate whether PCSK9 inhibitors could reduce the risk of CA-AKI in patients with ASCVD and evaluate their effectiveness under different disease types and risk stratifications.

## 2 Materials and methods

### 2.1 Ethical statement

The Institutional Human Research Committee of Tianjin Chest Hospital Ethics Committee (approval number: 2021LW-004) approved the study protocol, and the requirement for informed consent was waived because of its retrospective nature.

### 2.2 Patient population

Patients who underwent PCI at Tianjin Chest Hospital between January 2020 and December 2021 were enrolled in this retrospective-matched cohort study. The inclusion criteria were an age of 18–90 years and an ASCVD diagnosis. The enrolled patients received dual antiplatelet therapy, comprising aspirin and ticagrelor or clopidogrel, as well as statin-mediated lipid-lowering treatment. All study participants underwent standardized hydration therapy, and patients treated with evolocumab met the therapeutic criteria for evolocumab administration. Patients who met any of the following criteria were excluded from this study: contraindications to evolocumab usage, a requirement for renal replacement therapy, active infection, malignant tumor diagnosis, severe liver dysfunction (Child–Pugh class C), acute stroke history, or concurrent participation in another clinical trial. Women of childbearing age during screening or randomization, specifically those who had not undergone surgical sterilization or reached menopause, as well as breastfeeding women, were also deemed ineligible for inclusion in this study. Patients who were lost to follow-up were also excluded from the analysis. Standard PCI procedures were conducted, and the contrast agents were selected based on the patient's clinical status and the operator's discretion, without specific exclusions. Patients were categorized into the evolocumab and control groups based on evolocumab administration. All patients in the evolocumab group received a subcutaneous injection of 140 mg of evolocumab within 2 days before undergoing PCI.

### 2.3 Exposure

The exposure factor was the pre-PCI application of evolocumab. Laboratory tests were performed after patients with ASCVD were admitted to confirm the indications for PCSK9 inhibitors, and a subcutaneous injection of 140 mg of evolocumab was administered pre-PCI.

### 2.4 Sample size estimation

The exposed and control groups refer to the intervention groups receiving pre-PCI administration of evolocumab and those not receiving evolocumab, respectively. Additionally, the primary outcome measure was the cumulative incidence of CA-AKI. Based on our previous study on patients with acute myocardial infarction, the anticipated incidence rate was 6.7% and 20% in the exposed and control groups, respectively (10). Sample size calculations with a two-sided α of 0.05 and power of 90% yielded a requirement of 171 study participants for each group. Finally, a minimum of 186 study participants was deemed necessary for both groups, including at least 376 study participants, considering the potential loss to follow-up and refusal to participate (10%).

### 2.5 Control of missing data

A subset of patients was excluded because of missing data. Random sampling from the control group was conducted to address the potential bias arising from these exclusions by selecting patients with complete data at a 1:1 ratio to minimize bias. This sampling strategy was designed to reduce potential biases, thereby improving the study's reliability and applicability. Furthermore, using randomized sampling within the control group enables a more rigorous comparison and evaluation of the differences between the intervention and control groups, yielding more robust study findings.

### 2.6 Endpoint and definition of the study

This study's primary endpoint was CA-AKI occurrence. CA-AKI was defined as an absolute increase in blood creatinine concentration by 0.5 mg/dl (44.2 μmol/L) or 25% within 48 h of contrast exposure, indicating any AKI that occurred within 48 h of contrast administration (11). During hospitalization, the baseline was defined as the highest blood creatinine concentration within the 7 days preceding contrast exposure and before the initiation of standard hydration therapy. The highest blood creatinine concentration within 48 h after contrast exposure was used as the follow-up creatinine concentration to assess CA-AKI incidence. ASCVD was defined according to the criteria outlined in the 2018 American College of Cardiology/American Heart Association guidelines for lipid management (12).

Anemia was defined as plasma hemoglobin (HB) levels of <13 and <12 g/dl in males and females, respectively (13). Furthermore, the glomerular filtration rate was uniformly calculated using the Chronic Kidney Disease Epidemiology Collaboration formula.

### 2.7 Statistical analyses

Data analyses were performed using IBM SPSS Statistics for Windows, version 25.0 (IBM Corp., Armonk, NY, USA), and PASS (version 11; NCSS, LLC, Kaysville, UT, USA). Continuous variables are expressed as mean and standard deviation, and the *t-*test was performed for normally distributed continuous variables. The rank sum (Mann–Whiney *U*-test) and chi-square tests were performed for categorical variables.

The evolocumab and control groups were matched 1:1 using propensity-score matching to adjust for the influence of bias and confounding factors. We set the propensity-score matching caliper to 0.02 and used logistic regression analysis to evaluate the protective effect of evolocumab on CA-AKI. Statistical significance was set at a two-tailed *p*-value of <0.05. Based on univariate regression analysis of baseline data, considering a *p*-value of <0.2, the variables requiring adjustment were age, body mass index, emergency PCI, type II diabetes mellitus, hypertension, old myocardial infarction (OMI), usage of intra-aortic balloon pump (IABP), unstable angina, ST-elevation myocardial infarction (STEMI), non-ST-elevation myocardial infarction (NSTEMI), basal glucose, left ventricular ejection fraction (LVEF), HB, LDL, volume of contrast, and estimated glomerular filtration rate (eGFR).

**Figure 1:**
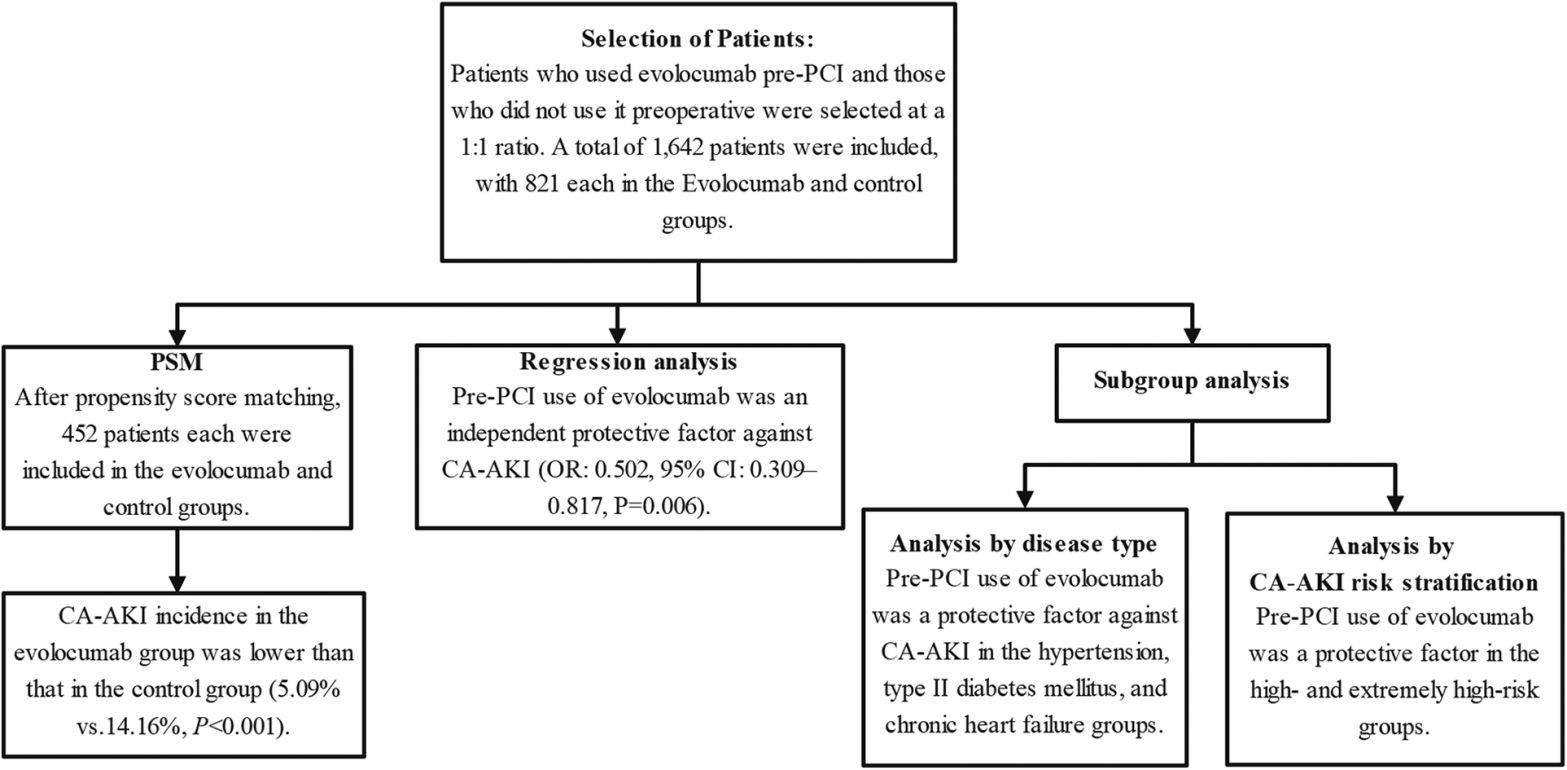
Research flowchart. The chart depicts the rigorous methodology employed in this study. The chart highlights post-propensity-score matching counts and pivotal outcomes, revealing that pre-PCI use of evolocumab is an independent protective factor against CA-AKI. Subgroup analyses further underscore the protective role of evolocumab in specific conditions, such as hypertension, type II diabetes mellitus, and chronic heart failure, and within certain CA-AKI risk strata. CA-AKI, contrast-associated acute kidney injury; PCI, percutaneous coronary intervention; PSM, propensity-score matching.

Furthermore, we performed subgroup regression analyses using two different grouping methods to investigate the medication's protective effect on CA-AKI. Specifically, the first method involved subgroup analysis based on various concomitant risk factors, including hypertension, type II diabetes mellitus, advanced age, body mass index, and the use of an IABP, whereas the second method was based on risk stratification of patients with CA-AKI (1). Figure 1 shows this study's overall methodology and patient selection process.

## 3 Results

### 3.1 Patient characteristics

Overall, 1,642 patients were included in this study. Of these, 821 patients each were in the evolocumab and control groups. The mean age of patients in the evolocumab group was 63.18 ± 9.60 years, with 565 (68.82%) being male, whereas that of those in the control group was 59.88 ± 11.46 years, with 547 (66.63%) being male. No statistically significant differences were found between the preoperative blood creatinine levels of patients in the evolocumab and control groups. Due to the absence of statistically significant differences in preoperative blood creatinine levels between the two patient groups, we did not conduct further subgroup analysis based on the presence or absence of chronic kidney disease. However, a statistically significant difference was found in the baseline eGFR levels between the two groups. Subsequently, we performed propensity-score matching on the data to minimize bias. The patients in both groups underwent a standard hydration protocol pre- and post-PCI procedures. We incorporated data based on the risk factors mentioned in the CA-AKI risk score previously published (1); therefore, differences in the number of coronary artery lesions, severity, and the quantity of percutaneous transluminal coronary angioplasty/stents were excluded from this analysis (1). Significant differences were observed in age, body mass index, hypertension, OMI, IABP, unstable angina, STEMI, NSTEMI, volume of contrast, eGFR, LVEF, HB, and LDL between the evolocumab and control groups. However, these differences between the two groups decreased after matching. Furthermore, a 1:1 propensity-score matching was performed, and 452 patients each were matched in the control and evolocumab groups. After matching, these differences between the two groups were reduced. Table 1 presents the baseline data for each group.

**Table 1:** Baseline characteristics before and after propensity-score matching.

Characteristic	Evolocumab group (*n* = 821)	Control group (*n* = 821)	Matched evolocumab group (*n* = 452)	Matched control group (*n* = 452)	*p_1_*	*p_2_*
Mean age (years)	63.18 ± 9.60	59.88 ± 11.46	61.50 ± 10.65	62.06 ± 10.24	<0.001	0.419
Distribution						
>75 years	63 (7.67%)	67 (8.16%)	41 (9.07%)	32 (7.08%)	0.715	0.272
≤75 years	758 (92.33%)	754 (91.84%)	411 (90.93%)	420 (92.92%)		
Sex (%)						
Male	565 (68.82%)	547 (66.63%)	303 (67.04%)	304 (67.26%)	0.342	0.944
Female	256 (31.18%)	274 (33.37%)	149 (32.96%)	148 (32.74%)		
Body mass index (kg/m^2^)	26.46 ± 3.79	25.49 ± 3.79	26.05 ± 3.23	26.03 ± 3.93	<0.001	0.929
Emergency PCI	43 (5.24%)	33 (4.02%)	21 (4.65%)	26 (5.75%)	0.240	0.454
Type II diabetes mellitus	283 (34.47%)	297 (36.18%)	168 (37.17%)	171 (37.83%)	0.470	0.837
Hypertension	521 (63.46%)	590 (71.86%)	307 (67.92%)	308 (68.14%)	<0.001	0.943
OMI	314 (38.25%)	198 (24.12%)	145 (32.08%)	147 (32.52%)	<0.001	0.887
IABP	27 (3.29%)	11 (1.34%)	7 (1.55%)	10 (2.21%)	0.009	0.463
UA	437 (52.23%)	664 (80.88%)	305 (67.48%)	312 (69.03%)	<0.001	0.617
SAP	2 (0.24%)	0 (0.00%)	1 (0.22%)	0 (0.00%)	0.500	0.239
STEMI	180 (21.92%)	63 (7.76%)	65 (14.38%)	60 (13.27%)	<0.001	0.630
NSTEMI	200 (24.36%)	105 (12.79%)	88 (19.47%)	85 (18.81%)	<0.001	0.800
CHF	818 (99.63%)	814 (99.15%)	450 (99.56%)	445 (98.45%)	0.205	0.085
Volume of contrast	150.24 ± 41.10	134.19 ± 41.90	142.15 ± 40.22	145.18 ± 43.48	<0.001	0.277
Creatinine level	76.73 ± 18.31	77.35 ± 24.53	77.61 ± 19.56	76.65 ± 18.15	0.565	0.443
eGFR	91.10 ± 19.81	88.14 ± 19.03	88.94 ± 19.54	89.18 ± 18.55	0.002	0.850
Basal glucose	6.70 ± 2.40	6.66 ± 2.65	6.71 ± 2.36	6.83 ± 2.75	0.737	0.505
LVEF	55.86 ± 8.35	56.82 ± 8.93	56.75 ± 8.234	56.88 ± 8.93	0.024	0.822
HB	13.97 ± 1.72	13.53 ± 1.60	13.62 ± 1.64	13.58 ± 1.65	0.001	0.696
LDL	3.26 ± 1.15	2.59 ± 0.95	2.91 ± 1.03	2.89 ± 1.01	<0.001	0.753

CHF, chronic heart failure; eGFR, estimated glomerular filtration rate; Emergency PCI, emergency percutaneous coronary intervention; HB, hemoglobin; IABP, intra-aortic balloon pump; LDL, low-density lipoprotein; LVEF, left ventricular ejection fraction; NSTEMI, non-ST-segment elevation myocardial infarction; OMI, old myocardial infarction; SAP, stable angina pectoris; STEMI, ST-segment elevation myocardial infarction; UA, unstable angina; *p_1_*, evolocumab group vs. control group; *p_2_*, matched evolocumab group vs. matched control group.

### 3.2 Adverse events analysis in patients receiving evolocumab

In terms of adverse events, we thoroughly reviewed data on adverse reactions experienced by patients in both groups, particularly those potentially attributable to evolocumab. Common adverse reactions listed on the evolocumab product label include nasal congestion, upper respiratory tract infections, influenza, back pain, injection site reactions, cough, urinary tract infections, sinusitis, headache, myalgia, dizziness, musculoskeletal pain, hypertension, diarrhea, and gastroenteritis. However, upon review of the data, none of these adverse reactions were reported in the evolocumab group, and there were no instances of allergic reactions to evolocumab observed.

### 3.3 Association between evolocumab and CA-AKI

The primary endpoint of CA-AKI was observed in 117 (7.13%) of the 1,642 patients. CA-AKI incidence was lower in the evolocumab group than in the control group (6.21% vs. 8.04%; *p *= 0.15). Patients in the evolocumab group had older age, higher body mass index, a greater prevalence of a history of OMI, a higher proportion using IABP, more cases of acute myocardial infarction, and higher contrast agent doses than those in the control group. However, the CA-AKI incidence was lower in the evolocumab group than in the control group. Therefore, we posit that this discrepancy is partly attributed to a potential protective effect of preoperative evolocumab usage against CA-AKI. It may also be associated with potential confounding factors and biases. We used propensity-score analysis to rigorously adjust for baseline discrepancies between the two groups in evaluating CA-AKI outcomes to minimize selection bias and potential confounders. Additionally, we established a caliper of 0.02 for propensity-score matching, after which significant differences were observed between the evolocumab and control groups (5.09% vs. 14.16%; *p *< 0.001). However, the postoperative serum creatinine level did not differ significantly between the two groups, with or without matching (Table 2).

**Table 2:** Incidence of CA-AKI in the evolocumab and control groups.

Outcome	Evolocumab group (*n* = 821)	Control group (*n* = 821)	Matched evolocumab group (*n* = 452)	Matched control group (*n* = 452)	*p_1_*	*p_2_*
CA-AKI	51 (6.21%)	66 (8.04%)	23 (5.09%)	64 (14.16%)	0.150	<0.001

CA-AKI, contrast-associated acute kidney injury; *p_1_*, evolocumab group vs. control group; *p_2_*, matched evolocumab group vs. matched control group.

**Figure 2:**
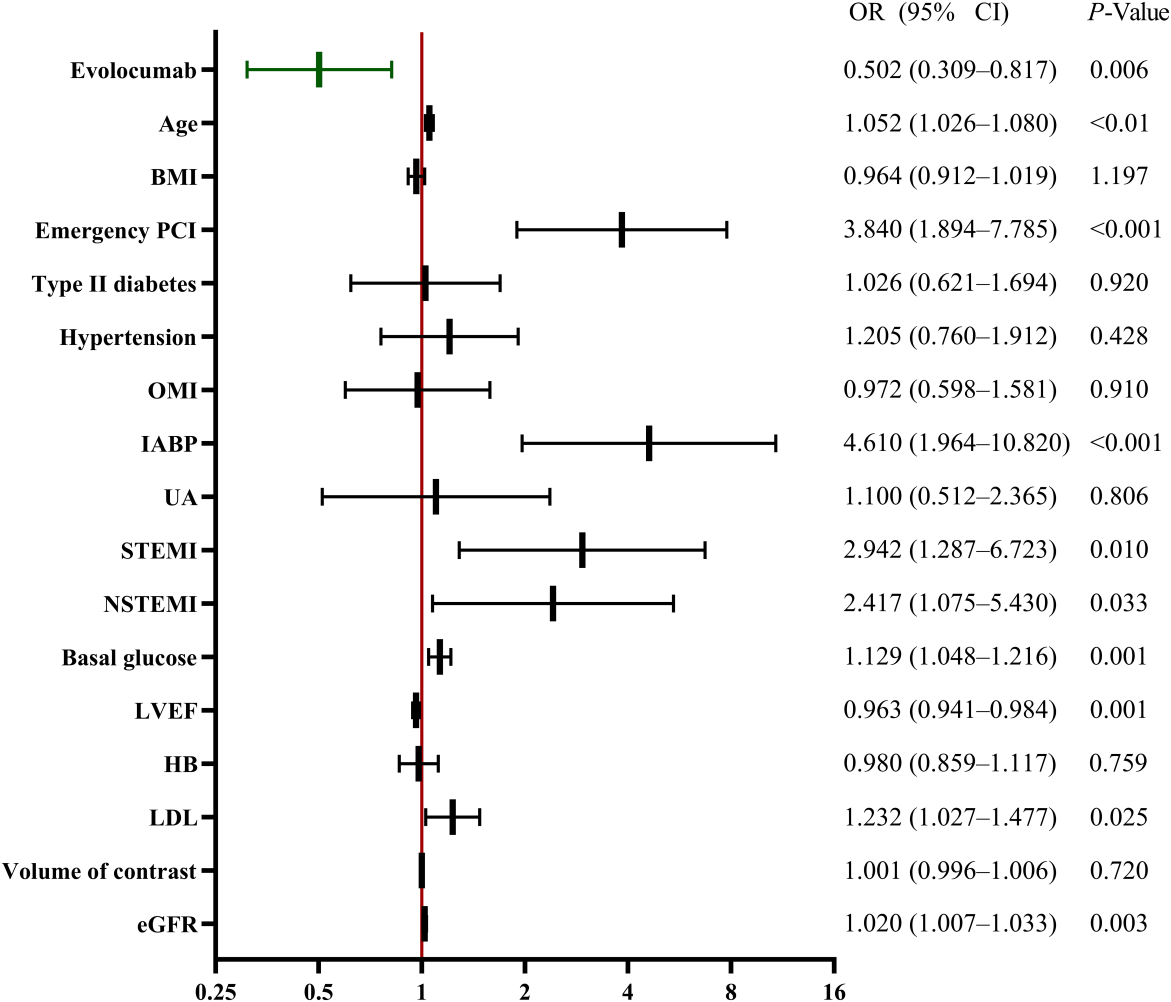
Multivariable analysis for CA-AKI predictors. Multivariable analysis showing various predictors of CA-AKI. Evolocumab was an independent protective factor against CA-AKI. Other factors, such as age, emergency PCI, IABP, STEMI, NSTEMI, basal glucose, LVEF, LDL, and eGFR, were also significant predictors of CA-AKI development. BMI, body mass index; CA-AKI, contrast-associated acute kidney injury; CI, confidence interval; EF, ejection fraction; HB, hemoglobin; IABP, intra-aortic balloon pump; LDL, low-density lipoprotein; NSTEMI, non-ST-segment elevation myocardial infarction; OMI, old myocardial infarction; PCI, percutaneous coronary intervention; STEMI, ST-segment elevation myocardial infarction; UA, unstable angina.

Multivariate regression analysis also revealed that evolocumab (odds ratio: 0.502, 95% confidence interval: 0.309–0.817, *p *= 0.006) was an independent protective factor for CA-AKI and that age, emergency PCI, IABP, STEMI, NSTEMI, basal glucose level, LVEF, LDL, and eGFR were independent predictors of CA-AKI development (Figure 2).

### 3.4 Subgroup analysis

**Figure 3:**
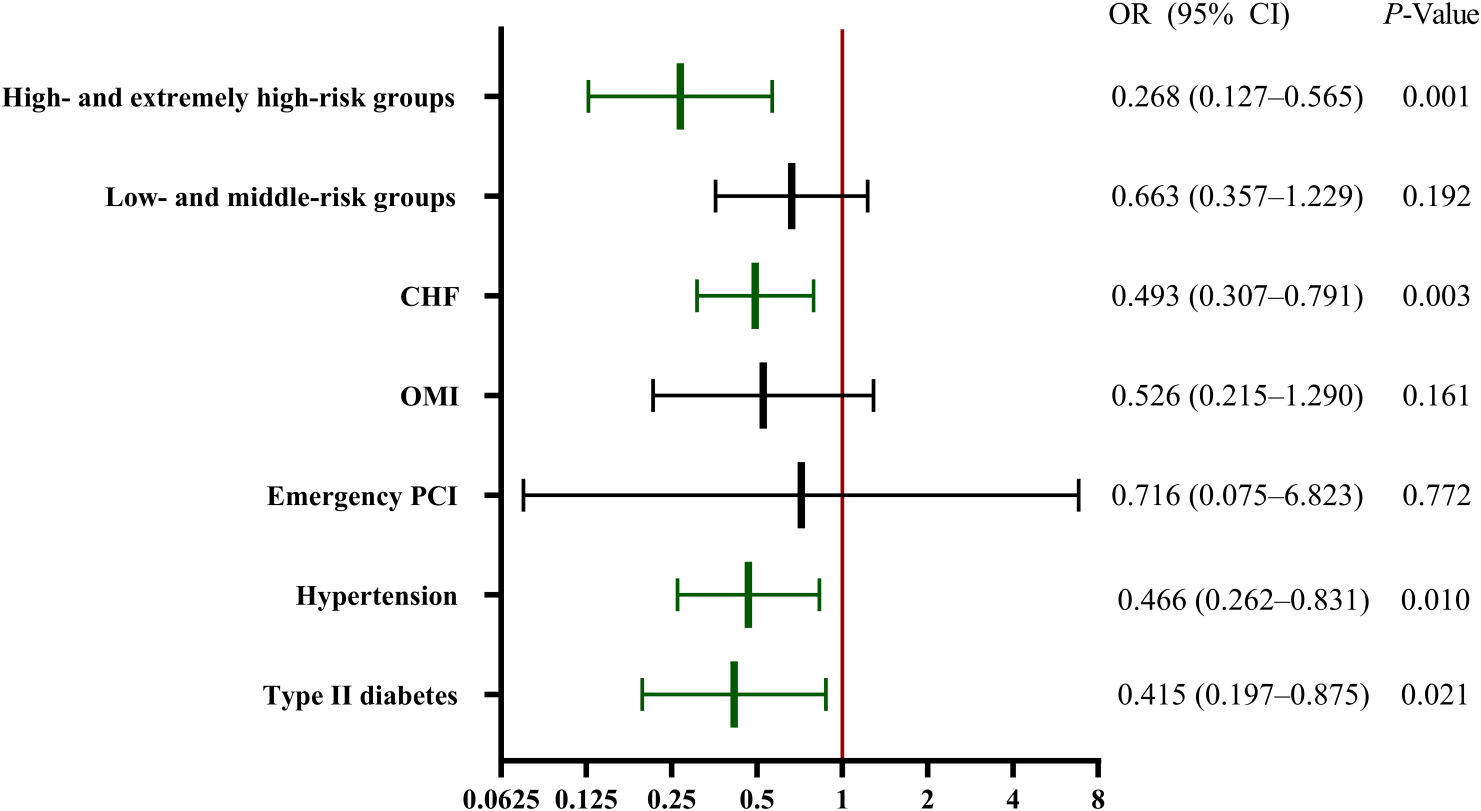
Subgroup regression analysis of CA-AKI. The efficacy of evolocumab in preventing CA-AKI in the various subgroups. Significant reductions in CA-AKI incidence were observed in the hypertension, type II diabetes mellitus, and CHF groups. However, the emergency PCI and OMI groups did not exhibit significant reductions. A further stratified risk analysis highlighted a significant protective effect of evolocumab, primarily in the high- and extremely high-risk cohorts. CA-AKI, contrast-associated acute kidney injury; CHF, chronic heart failure; CI, confidence interval; OMI, old myocardial infarction; OR, odds ratio; PCI, percutaneous coronary intervention.

Figure 3 illustrates the efficacy of evolocumab in preventing CA-AKI in these subgroups. A significant reduction was observed in CA-AKI in the hypertension, type II diabetes mellitus, and chronic heart failure groups but not in the emergency PCI or OMI groups. Subgroup analysis was further conducted on patients based on a contemporary simple risk score for CA-AKI prediction pre-PCI, as previously published (1). We combined the low- and middle-risk populations and the high- and extremely high-risk groups into respective single groups because of the limited number of patients in the low- and extremely high-risk groups. The results showed a significant protective effect of evolocumab in the high- and extremely high-risk groups (Figure 3).

## 4 Discussion

### 4.1 CA-AKI

CA-AKI is a leading cause of new-onset acute renal insufficiency in hospitalized patients, considering the extensive use of PCI worldwide. Currently, it is treated with statins and hydration therapies. Studies have demonstrated that high-dose statins have no significant preventive effect against CA-AKI (14) and may even increase CA-AKI risk (15). Excessive hydration can also increase the risk of heart failure, arrhythmia, and short-term mortality in high-risk patients. Therefore, newly updated guidelines no longer recommend standardized hydration prophylaxis for most patients (16).

This is the first study to directly investigate the preventive and therapeutic effects of PCSK9 inhibitors on CA-AKI in patients with ASCVD. Our main findings reveal that administering evolocumab to patients with ASCVD before exposure to contrast agents reduces the risk of CA-AKI. Similar outcomes were also observed in the subgroups of patients with hypertension, type II diabetes mellitus, and chronic heart failure. Notably, evolocumab demonstrated significant protective effects against CA-AKI within the high- and extremely high-risk populations based on CA-AKI risk scores.

### 4.2 PCSK9 inhibitors and CA-AKI

Studies have shown that apoptosis, pyroptosis, and inflammatory responses play crucial roles in CA-AKI occurrence and development (17–21). Recent studies have demonstrated that PCSK9 inhibitors can reduce the extent of necroptosis, apoptosis, and inflammatory responses, thereby alleviating cell damage (22–27). Undoubtedly, PCSK9 inhibitor treatment provides a novel approach for CA-AKI prevention in high-risk populations. However, the association between the preoperative use of PCSK9 inhibitors and CA-AKI incidence in patients undergoing PCI has not been explored.

Our study shows that administering evolocumab before contrast exposure can effectively mitigate CA-AKI risk. Therefore, we rigorously adjusted the baseline metrics using propensity-score analysis for the evolocumab and control groups to minimize the influence of selection bias and potential confounding variables. Propensity-score matching revealed more significant differences between the evolocumab and control groups. Multiple logistic regression was used to confirm the protective effect of evolocumab after adjusting for various confounding factors, including age, body mass index, type II diabetes mellitus, hypertension, history of myocardial infarction, emergency PCI, IABP, and combination medications. The findings showed that evolocumab was stable in reducing CA-AKI risk. Subsequent subgroup analyses performed based on various diseases revealed that evolocumab significantly reduced CA-AKI incidence in individuals with hypertension, type II diabetes mellitus, and chronic heart failure.

More importantly, a subgroup analysis was conducted based on the previously published risk stratification of CA-AKI, which revealed the significant protective effect of evolocumab in the high- and extremely high-risk groups (1).

### 4.3 Mechanism

CA-AKI onset has been associated with the activation of inflammatory responses, oxidative stress, apoptosis, and pyroptosis despite the unknown precise mechanism underlying the protective effect of evolocumab against CA-AKI. Recent studies have established a strong association between these factors and PCSK9 inhibitors (2).

PCSK9 belongs to the proprotein convertase family and comprises a set of serine proteases, with the liver as its primary origin. However, it is also found in extrahepatic tissues, including the kidneys, small intestine, brain, heart, and blood vessels.

Studies have also shown a significant association between PCSK9 and the inflammatory response. PCSK9 acts as a pro-inflammatory mediator, and its overexpression leads to vascular inflammation (28). Additionally, elevated serum PCSK9 levels have been found in individuals with systemic inflammatory response syndromes and sepsis. PCSK9 overexpression also augmented the systemic release of interleukin (IL)-6 and exacerbated the visceral inflammatory response (26, 29). However, its deficiency is associated with reduced circulating levels of IL-6 and improved organ inflammation (29, 30). Significant correlations were found between serum PCSK9 concentrations and pro-inflammatory cytokines, IL-1β, tumor necrosis factor-alpha, macrophage colony-stimulating factor, and high-sensitivity C-reactive protein (26). PCSK9 can augment the production of pro-inflammatory cytokines, and specific investigations have revealed the toll-like receptor 4/nuclear factor kappa B (TLR4/NF-κB) signaling pathway as a principal affected pathway, mediating the consequences of PCSK9-induced elevation of pro-inflammatory cytokines (27). A reciprocal amplification loop exists between reactive oxygen species generation in the mitochondria and PCSK9 expression. The induction of lectin-like oxidation of LDL receptor-1 by reactive oxygen species may also facilitate its interaction with PCSK9, leading to oxidative stress, inflammation, and injury to renal tubular epithelial cells (26, 27). Lectin-like oxidation of LDL receptor-1 is a scavenger receptor for oxidized LDL cholesterol and can be induced in glomerular mesangial cells (31). Furthermore, inhibiting the PCSK9 protein in endothelial cells can effectively decrease lectin-like oxidation of LDL receptor-1 and reactive oxygen species expression, thereby mitigating inflammation and oxidative stress responses and reducing the risk of CA-AKI.

The apoptosis of vascular and renal tubular epithelial cells influences CA-AKI development. PCSK9 is significantly associated with inflammation and apoptosis progression in atherosclerosis; therefore, inhibiting its expression may reduce apoptosis and decrease CA-AKI incidence (32).

Moreover, Tang et al. (27) reported that silencing the *PCSK9* gene can alleviate intracellular inflammatory responses in macrophages by suppressing the TLR4/NF-κB signaling pathway, directly inhibiting atherosclerosis. Kong et al. (24) found that inhibiting the TLR4/NF-κB signaling pathway in macrophages can decrease NOD-like receptor pyrin domain-containing-3 (NLRP3) expression and suppress the activation of caspase-1, reducing IL-1β production and lowering cellular pyroptosis level. Wang et al. (25) discovered that PCSK9 activates the NLRP3 inflammasome signaling pathway (NLRP3, apoptosis-associated speck-like protein containing a C-terminal caspase recruitment domain, caspase-1, IL-1β, and IL-18), subsequently inducing caspase-1-dependent cellular pyroptosis. Silencing the *PCSK9* gene significantly inhibits the NLRP3 inflammasome signaling expression, gasdermin D-N-terminal cleavage product, and lactate dehydrogenase release. However, patients with chronic myocardial ischemia show highly elevated levels of serum PCSK9, NLRP3 inflammasome signaling, and cellular pyroptosis (gasdermin D and lactate dehydrogenase release). As previously mentioned, these studies suggest that silencing the *PCSK9* gene downregulates NLRP3 expression, thereby reducing cellular pyroptosis levels.

### 4.4 Clinical implications

This study showed a significant association between the preoperative administration of PCSK9 inhibitors in PCI and a reduced CA-AKI incidence after adjusting for relevant risk factors. Additionally, this protective effect was evident in the subgroups of patients with hypertension, type II diabetes mellitus, and chronic heart failure. Importantly, PCSK9 inhibitors exhibited a more pronounced protective effect against CA-AKI in high- and extremely high-risk patient groups than in the low- and middle-risk groups.

### 4.5 Study limitations

Despite efforts to address potential biases, our retrospective, non-randomized design may have introduced inherent limitations, which could affect the robustness and generalizability of our findings. While propensity-score matching was utilized, residual confounding factors may still have influenced the observed outcomes. Additionally, our study was conducted at a single center, potentially limiting the broader applicability of our results when compared to those from multicenter studies. Furthermore, the effects of other renoprotective agents or concomitant medications were not extensively explored, which may have impacted our findings. Future research should include larger-scale, multicenter, randomized controlled trials to minimize confounding and validate the protective effects of PCSK9 inhibitors on CA-AKI. Basic biology research is also warranted to elucidate the underlying mechanisms of action. Moreover, it would be beneficial to conduct a cost-effectiveness analysis to assess the financial implications of incorporating evolocumab into the standard of care for preventing CA-AKI, which was not addressed in our study. Finally, exploring the long-term outcomes, including overall survival and cardiovascular events, in patients receiving evolocumab compared to standard treatment would provide valuable insights into the clinical benefits of this intervention.

## 5 Conclusions

This study demonstrated the efficacy of PCSK9 inhibitors in mitigating CA-AKI incidence. Specifically, this finding was consistent across the subgroups of patients with type II diabetes mellitus and hypertension, indicating that PCSK9 inhibitors serve as a protective factor against CA-AKI. Furthermore, the risk stratification analysis revealed a significantly greater protective effect of PCSK9 inhibitors in high- and extremely high-risk groups than in low- and middle-risk groups, providing novel insights and approaches for preventing CA-AKI in high-risk individuals.

## Data Availability

The raw data supporting the conclusions of this article will be made available by the authors, without undue reservation.
